# U-Net based vessel segmentation for murine brains with small micro-magnetic resonance imaging reference datasets

**DOI:** 10.1371/journal.pone.0291946

**Published:** 2023-10-12

**Authors:** Christoph Praschl, Lydia M. Zopf, Emma Kiemeyer, Ines Langthallner, Daniel Ritzberger, Adrian Slowak, Martin Weigl, Valentin Blüml, Nebojša Nešić, Miloš Stojmenović, Kathrin M. Kniewallner, Ludwig Aigner, Stephan Winkler, Andreas Walter

**Affiliations:** 1 Department of Medical and Bioinformatics, School of Informatics, Communications and Media, University of Applied Sciences Upper Austria, Hagenberg i. M., Austria; 2 Ludwig Boltzmann Institute for Experimental and Clinical Traumatology in the AUVA trauma research center, Austrian Cluster for Tissue Regeneration, Vienna, Austria; 3 Vienna BioCenter Core Facilities GmbH (VBCF), Vienna, Austria; 4 Faculty of Informatics and Computation, Singidunum University, Belgrade, Serbia; 5 Institute of Molecular Regenerative Medicine, Spinal Cord Injury and Tissue Regeneration Center Salzburg, Paracelsus Medical University, Salzburg, Austria; 6 Centre of Optical Technologies, Aalen University, Aalen, Germany; Islamia University of Bahawalpur: The Islamia University of Bahawalpur Pakistan, PAKISTAN

## Abstract

Identification and quantitative segmentation of individual blood vessels in mice visualized with preclinical imaging techniques is a tedious, manual or semiautomated task that can require weeks of reviewing hundreds of levels of individual data sets. Preclinical imaging, such as micro-magnetic resonance imaging (μMRI) can produce tomographic datasets of murine vasculature across length scales and organs, which is of outmost importance to study tumor progression, angiogenesis, or vascular risk factors for diseases such as Alzheimer’s. Training a neural network capable of accurate segmentation results requires a sufficiently large amount of labelled data, which takes a long time to compile. Recently, several reasonably automated approaches have emerged in the preclinical context but still require significant manual input and are less accurate than the deep learning approach presented in this paper—quantified by the Dice score. In this work, the implementation of a shallow, three-dimensional U-Net architecture for the segmentation of vessels in murine brains is presented, which is (1) open-source, (2) can be achieved with a small dataset (in this work only 8 μMRI imaging stacks of mouse brains were available), and (3) requires only a small subset of labelled training data. The presented model is evaluated together with two post-processing methodologies using a cross-validation, which results in an average Dice score of 61.34% in its best setup. The results show, that the methodology is able to detect blood vessels faster and more reliably compared to state-of-the-art vesselness filters with an average Dice score of 43.88% for the used dataset.

## Introduction

To address biomedical research questions across multiple scales, the frontiers of bioimaging are currently being pushed toward integrating and correlating several modalities. In particular, in-vivo imaging is being integrated with ex-vivo microscopy in order to bridge the gap between preclinical and biological imaging. Due to its ability to acquire three-dimensional (3D) high-resolution datasets without compromising the integrity of specimens, micro-Magnetic Resonance Imaging (μMRI) is a powerful imaging technique for preclinical imaging. At a resolution of a few micrometers, MRI gathers metabolic, physiological, and functional information. Due to its non-ionizing interaction with the net nuclear magnetic moment of nucleons, in contrast to μCT, repeated studies are feasible, with no known side effects, to monitor physiological and pathophysiological processes in-vivo, including ageing or disease progression. For preclinical in-vivo studies, murine animal models still represent the state-of-the-art for multiple areas [1–4]. Magnetic resonance angiography (MRA) plays a crucial role in monitoring preclinical in vivo studies with murine model organisms, and specifically to qualitatively analyze murine vasculature in brains or tumors [5–8].

Increasingly, raw images of several gigabytes per imaging session are collected due to advances in bioimaging and preclinical imaging. There is an exponential growth in the volume of data collected in biomedical imaging, including MRI, and the field is entering a world of big data. Increasing throughput and automation will generate even more data, raising the pivotal question of how to manage and analyze them.

One important goal of these preclinical studies in mice is to quantitatively annotate and segment the acquired volume stacks to quantify vascular morphology and gather important insights into the mechanisms of cerebrovascular disease. For example, cerebrovascular dysfunction leads to neurodegeneration and approximately 60–90% of Alzheimer disease patients show cerebrovascular pathologies. Despite many advances in automatic vasculature quantification mainly based on machine-learning approaches, murine blood vessels are often still segmented semiautomatically or manually to extract quantitative information—with recent applications of neural networks for automated data processing mainly restricted to human radiology [9]. Machine learning approaches at the segmentation of vascular morphology requires training datasets [10], which leads to a chicken-egg problem, since many in-vivo studies lack training data.

In fact, to the authors’ best knowledge, apart from implementation for advanced fluorescence microscopy [11], we are not aware of a single study that segments murine brain vasculature in a fully automated way using neural networks—despite the urgent need in the academic field to generate throughput and accelerate the process. In fact, manual segmentation can take several weeks if carried out by a single person—and modern segmentation schemes, such as vesselness filters, are often not applicable in mice or do not perform to their standards [12] due to the much smaller vessel diameters and lengths as compared to human angiography. Specifically, many automated segmentation approaches remain inaccessible to non-specialists, and can be of limited use on data for which they were not developed or trained. The evaluation of these imaging techniques requires time and cost intensive expert knowledge. For this reason, this work evaluates the utilization of a fully automated deep learning model based on the U-Net architecture as introduced by Ronneberger et al. [13] allowing to segment vessels of mice brains in the context of a small μMRI reference dataset. The imaging data was acquired in mice to evaluate cerebrovascular pathologies and their role in neurodegeneration and characterize these structural changes. Typical hallmarks of cerebrovascular dysfunction are fragmented vessels, changes in blood vessel diameter, or basement membrane thickening of capillaries—which we aimed at characterizing further. This requires the fast and automated segmentation of murine brain vasculature with a limited amount of available training sets. The presented methodology is compared to the segmentation approach using vesselness filters [14] and aims to tackle the following questions:

- *RQ*_1_: *Are deep learning architectures applicable for the segmentation of mice brain vessels using a small μMRI reference dataset?*
  It is examined if algorithms from the field of computer vision using deep learning architectures can be used to detect blood vessels in *μ*MRI data with only a small dataset for the model training.
- *RQ*_2_: *Which influence do different post-processing methods have?*
  The influence of multiple post-processing methods should be evaluated based on the segmentation confidences of the used deep learning model.
- *RQ*_3_: *How does a deep learning based methodology perform compared to classic approaches such as vesselness filters?*
  The results of both, the deep learning based methodology, as well as the utilization of state-of-the-art vesselness filters are compared to evaluate their usability in the context of brain vessel segmentations based on *μ*MRI data.

The remainder of this work outlines the background in the first section highlighting the motivation for an automated segmentation approach for μMRI image stacks, followed by the proposed approach including the used image acquisition process and a description of the dataset, as well as the actual U-Net based image processing modalities and an overview of the used setup. Followed by the methodology, the results are listed based on the model’s Dice Score and time measurements, which are discussed compared to a state-of-the-art vesselness filter. This is followed by the related work, which is shown based on a literature review in the context of vessel segmentations in brains. Finally, the work is concluded, answering the research questions and emphasizing the advantages of our approach.

## Background

The task of differentiating a wanted foreground (FG) information from the remaining background (BG) within a given image can be tackled using classical intensity-based segmentation approaches such as region growing [15], watershed transformations [16], as well as graph segmentations like the graph cut algorithm [17]. These algorithms share the need for some level of user interaction, e.g. in the form of seed points for the FG or BG. In the case of the former two algorithms, these seed points are used for the training of a Gaussian Mixture model, which represents the basis for the segmentation task. Next to these approaches, there are also unsupervised strategies like the KMeans or MeanShift [18] algorithm, that create results comparable to the watershed approach.

Additionally, also more sophisticated methodologies can be used, that also incorporate information of the searched object’s shape. For example, algorithms such as Active Shape Models [19], Level Set segmentation [20] or Statistical Shape Models use principal component analysis allowing to solve instance segmentation as a heuristic registration process applicable in various domains [21].

The mentioned algorithms are next to many others also used for in-vivo analysis based on the separation of vessels (FG) from the remaining tissue (BG) in the area of e.g. the abdomen or organs like liver or brain [22–30]. In addition to that, so called vesselness filters have been developed specifically for the domain of vessel segmentation and have been commonly used in this area over the last decades [23, 24, 27]. Recently, with convolutions as the key image processing function for convolutional neural networks (CNN), various machine learning based network and architecture types have pushed the topic of vessel segmentation forward.

Especially, in the area of retinal vessel segmentation, the utilization of 2D CNNs is widespread [31–33], but mainly restricted to human studies. Besides, similar approaches utilizing three-dimensional instead of two-dimensional information have been introduced, e.g., in the area of liver vessel segmentation [34–36]—again exclusively in the field of human radiology. All these studies are mainly limited to human blood vessel segmentations—and can only be applied in a limited way to preclinical murine models. This is mainly since, on average, brain blood vessels of mice amount only to half of the diameter of human vessels and are significantly shorter. The smallest blood vessels in the mouse brain, the capillaries, typically have diameters between 3 and 5 micrometers—which makes them difficult to visualize with high signal-to-noise ratio and to segment. For the segmentation of murine blood vessels, a high μMRI resolution is required, which leads to further problems, such as large data sets, long acquisition times and the risk of motion artefacts, either in living samples or due to lack of fixation of preserved samples. Even with well-fixed specimens, movement can occur during long scans due to temperature differences that cause the material to expand or shrink. To make matters worse, the number of available animals and data stacks are usually limited to less than 10 in preclinical studies carried out in an academic research context since we are morally obligated to minimize the number of animal studies. Hence, for a specific study, there are only a limited amount of training datasets available—which, again, restricts the use of automatic segmentations based on neural networks. Manual or semiautomatic segmentation of imaging data is challenging and time consuming. The obtained results can vary from one person to another, and even the availability of imaging software and graphic tablets can influence the outcome [6]. Automatic segmentation is often based on thresholding, which is extremely difficult especially for MRI images, because the values vary even within a stack and a brightness gradient is present: In the MRI dataset we used for this study, blood vessels are shown as black dots in a grey-value image. But not all black dots correspond to blood vessels; also cavities in the brain are shown in black and are sometimes only shown as small dots in a 2D slice. When segmenting manually, only by looking at the sections before and after the currently processed slice and with anatomical knowledge of an expert, the correct identification of blood vessels is possible. For automatic segmentation a tubeness filter (c.f. https://www.longair.net/edinburgh/imagej/tubeness/) as implemented for the software ImageJ [37] can be used to avoid such incorrect labelling and to improve the result. However, manual improvements are usually necessary [12], which in turn leads to the problem of human influence on the result and makes the segmentation process slow and tedious. Hence, our aim was to establish an open-access, reproducible workflow to (1) improve the segmentation of murine vasculature, (2) automate the process, and (3) ensure consistent scores even with a limited number of training data.

## Methods

This study was carried out in strict accordance with the recommendations in the Guide for the Care and Use of Laboratory Animals of Austrian Federal Ministry of Education, Science and Research. The protocol was approved by the National Ministry of the Federal Republic of Austria (Number: BMWFW-66.019/0011-WF/V/3b/2016, European Community Council Directive (86/609/EEC)). All efforts were made to minimize suffering.

**Fig 1:**
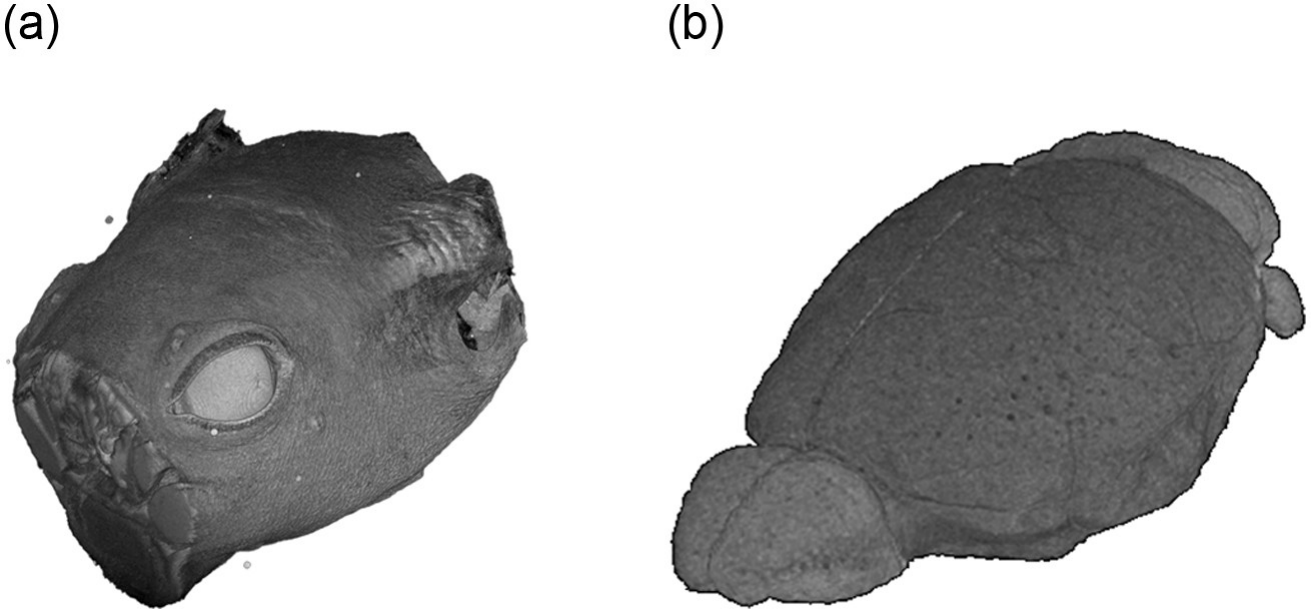
Sample image stack of the created dataset showing (a) the complete head of one mouse and (b) the segmented brain, visualized using the software MeVisLab [38]. The outer, background area was removed for visualization purposes.

In this work, a deep learning approach for the fully automatic segmentation of vessels in mice brains in the context of preclinical studies is presented. This task is addressed using a shallow U-Net segmentation model, which is trained on a small *μ*MRI reference dataset. One sample image stack of this dataset is shown in Fig 1.

### MRI & image acquisition

The methods presented in this work are implemented using a small reference dataset. For the μMRI data acquisition, animals were deeply anesthetized using a ketamine (20.38 mg/ml), xylazine (5.38 mg/ml) and acepromazine (0.29 mg/ml) mixture and transcardially perfused for immunohistochemistry as previously described [39, 40]. Following perfusion, the brains were extracted and post-fixed or immersion-fixed in 4% paraformaldehyde (PFA) in 0.1 M sodium phosphate buffer solution (pH = 7.4) for cryoprotection overnight at 4°C. To shorten longitudinal relaxation time (T1) for MRI imaging, heads were immerged in 1% gadolinium (ProHance^®^, Bracco, Germany) solution for 24 hours. Sagittal sections of 40 μm were cut on dry ice with a sliding microtome (Leica SM 2000R). During the scan, the heads were placed in a 50ml Falcon tube filled with Fomblin®. MR images were acquired with a 15.2 T Biospec horizontal bore scanner (Bruker BioSpin, Ettlingen, Germany), and BFG6S-100 actively shielded gradient system (750 mT/m maximum gradient strength). A quadrature transmit/receive volume coil (23 mm inner diameter, Bruker BioSpin) was used. A fast imaging with steady state precession (FISP) sequence was used with TR/TE 9/4.5 ms, 503 μm3 spatial resolution, 56 averages for all mice.

**Fig 2:**
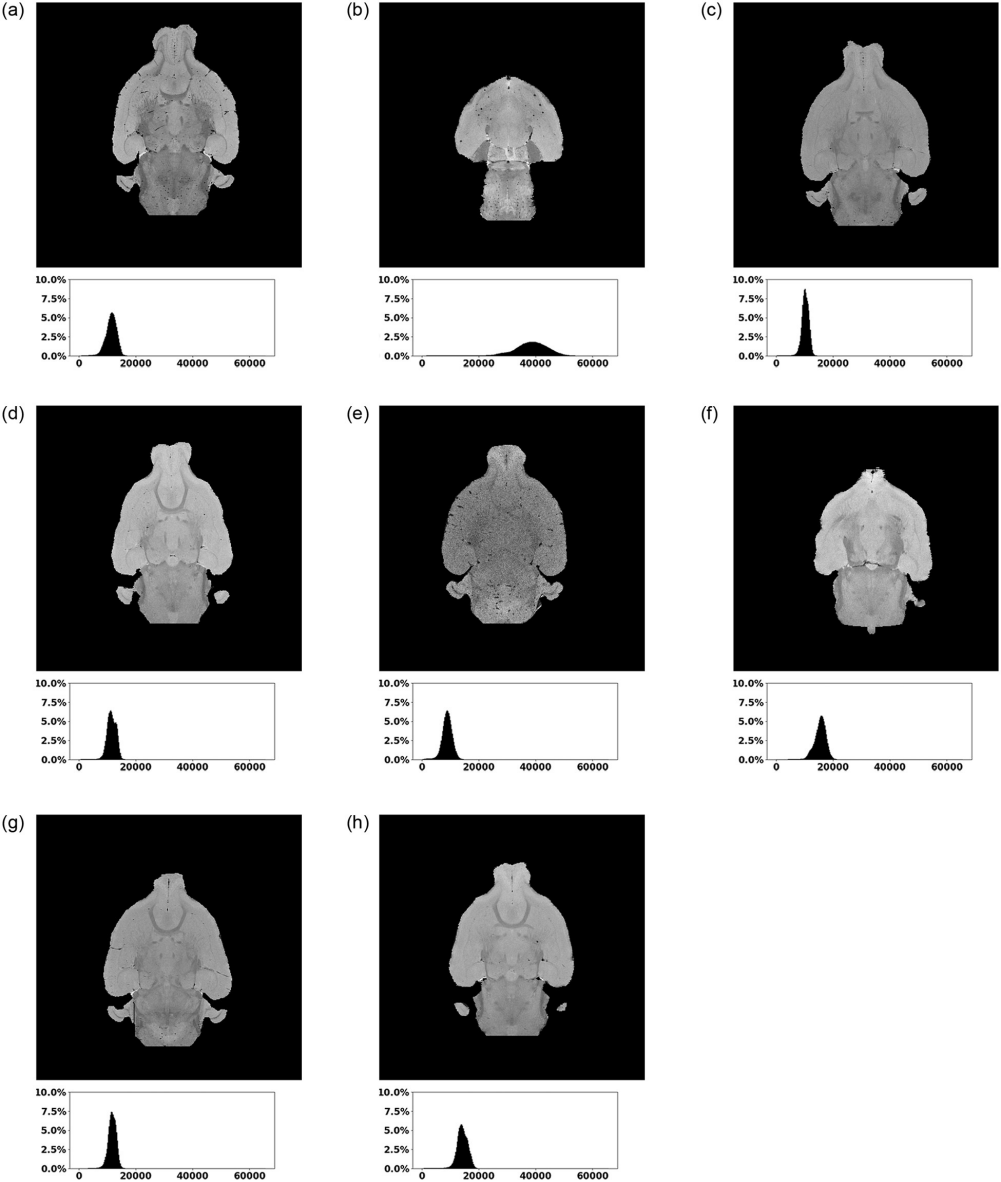
The eight mice image stacks of the dataset showing the wild type mice in (a) to (d) and the knockout types in (e) to (h), each represented by one masked layer (*l* = 200) and the associated histogram. The first two images per row show brains of adult (a) (b) (e) (f) and the remaining two of young mice (c) (d) (g) (h). The histogram shows the percentage value distribution within the brain area of the whole stack.

**Fig 3:**
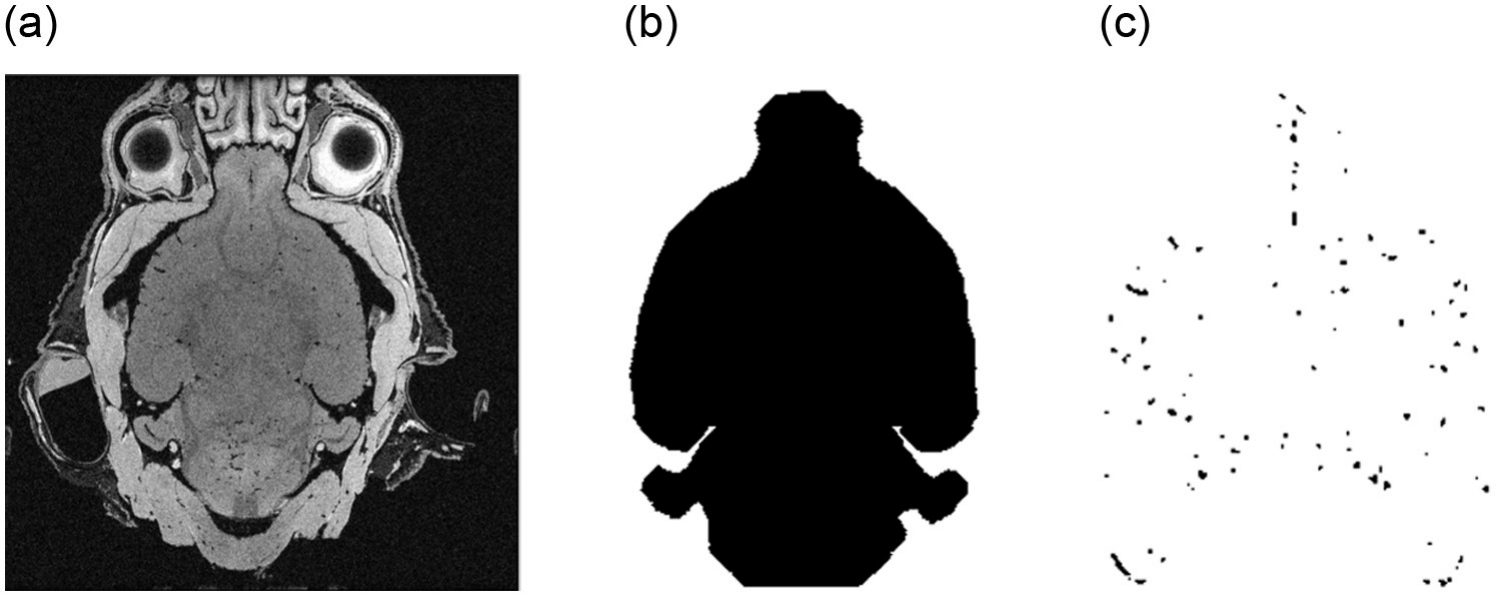
One sample layer of the image stack from Fig 1 with (a) the specific MRI layer (*l* = 200), (b) the associated brain mask and (c) the ground truth mask of the vessels.

The dataset consists of only eight μMRI image stacks with a size of 400 × 400 × 320 voxels and a value range of an unsigned integer [0, 65535], as shown in Fig 1. These images contain MRI scans of the heads from four wild- and four knockout-type mice (c.f. Fig 2) and have slightly deviating characteristics regarding the vertical offset, the level of noise and the brightness. Especially, Fig 2b highly differs from the remaining images with a higher mean value of 37761.69 as shown in Table 1. The stacks start and end with layers without any relevant information, i.e. that do not contain the actual head and brain information. This covers about the first 3% and the last 3% of the layers and is related to the MRI scan procedure. Based on the raw image stacks, two types of ground truth data are manually created. On the one hand, brain masks are prepared, allowing to restrict the presented segmentation approach on the brain itself. On the other hand, the actual ground truth masks for the blood vessels are also added to the dataset. The ground truth data is manually created using ImageJ [37] and its Tubeness plugin. The segmented vessels are morphologically transformed to removed artifacts. Finally, the created vessel masks are manually post-processed and improved using the Amira software [41]. Fig 3 shows one layer (*l* = 200) of a sample image stack with the associated brain mask and the ground truth vessels. The number of voxels representing blood vessels are distributed within a range of [0.036%, 0.134%] in the ground truth masks, which is also shown in Table 2. This distribution shows a strong scatter, with a proportion in three images over 0.12% and in five images with less than 0.06%. The different distribution can be led back to the manual labelling process, but partially also to the different ages of the animals, with four young and four older subjects.

**Table 1:** Value distribution of the masked image stacks (within the brain area) shown in Fig 2 within the created dataset.

	Stack 1 (Fig 2a)	Stack 2 (Fig 2b)	Stack 3 (Fig 2c)	Stack 4 (Fig 2d)	Stack 5 (Fig 2e)	Stack 6 (Fig 2f)	Stack 7 (Fig 2g)	Stack 8 (Fig 2h)
Min	38.00	9.00	6.00	35.00	6.00	43.00	24.00	20.00
Average	11247.10	37761.69	10165.85	11430.44	8800.10	15364.41	11625.19	14133.43
Max	24063.00	65535.00	23373.00	23834.00	20547.00	25315.00	24201.00	25504.00

**Table 2:** Distribution of foreground information (vessels) and background information within the image stacks of the created MRI dataset.

	Stack 1 (Fig 2a)	Stack 2 (Fig 2b)	Stack 3 (Fig 2c)	Stack 4 (Fig 2d)	Stack 5 (Fig 2e)	Stack 6 (Fig 2f)	Stack 7 (Fig 2g)	Stack 8 (Fig 2h)
Vessels	0.132%	0.127%	0.037%	0.040%	0.134%	0.044%	0.057%	0.036%
Background	99.868%	99.873%	99.963%	99.960%	99.866%	99.956%	99.943%	99.964%

### Image processing

**Fig 4:**
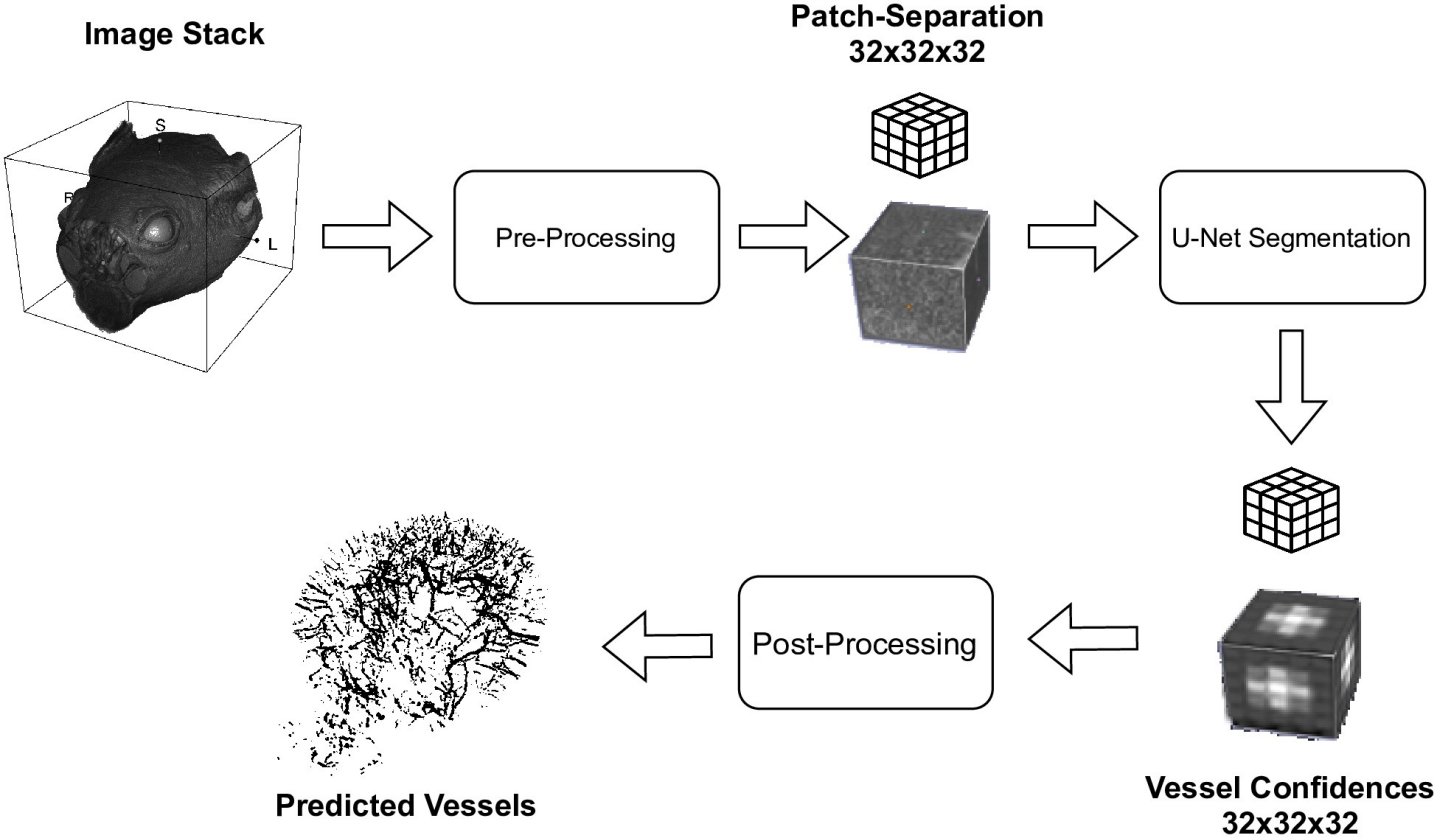
The overall process overview of the proposed methodology, used to extract blood vessels from MRI image stacks using an U-Net segmentation model.

The proposed methodology consists of three major parts: (I) the pre-processing of the input image stacks, (II) the U-Net based segmentation of the volume and (III) the post-processing of the segmentation confidences. This process is shown in Fig 4.

**Fig 5:**
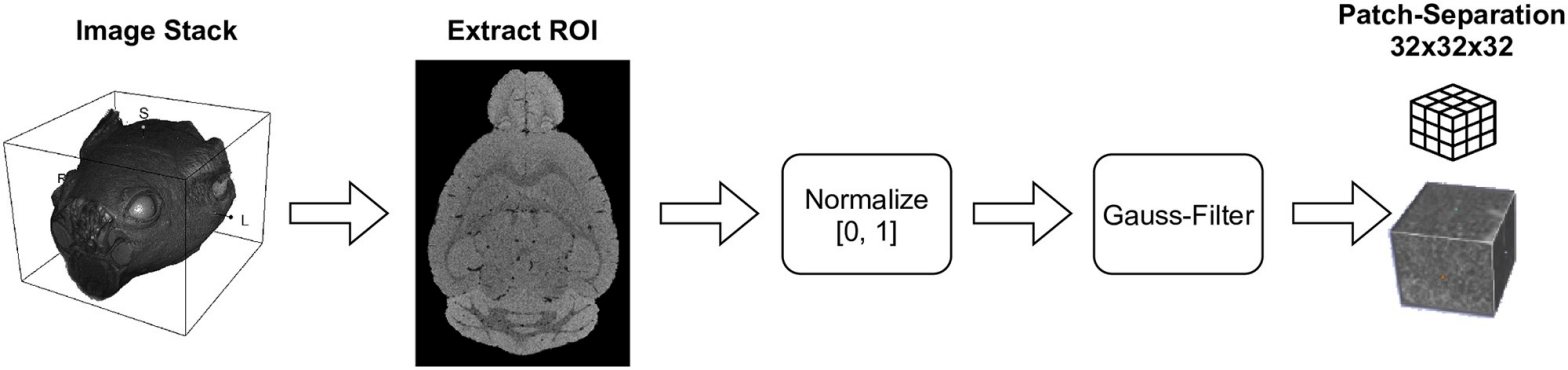
The pre-processing steps, that are applied to the individual image stacks as basis for the training of the segmentation model.

The used segmentation model is based on the U-Net architecture as introduced by Ronneberger et Al. [13], respectively on the adapted network structure for three-dimensional inputs as introduced by Çiçek et Al. [42]. This model is trained using the presented base dataset. Due to the characteristics of the image stacks within this dataset, a pre-processing is applied. This pre-processing consists of multiple consecutively steps (c.f. Fig 5). First, the region of interest (ROI) is extracted using the manually created brain masks, which are padded to the next bigger multiple of 32 in all dimensions. The so created ROIs are then blurred using a Gaussian kernel with *σ* = 0.5 to reduce the noise and additionally normalized to a range of [0, 1], which allows minimizing the influence of runaways like Stack 2 (c.f. Fig 2b). Finally, the images are split into patches with a size of 32 × 32 × 32 voxels. The individual results of the pre-processing steps are samplewise shown for one layer in Fig 8 in the S1 Appendix.

Using the created patches, a training- as well as a test-dataset are created with a ratio of 80% to 20%. The training dataset is further enlarged using multiple augmentation methods, which are randomly applied. Namely, a random flip along the x-, the y- or both axis, a normal distributed brightness adaption with *σ* = 0.1 and/or a random noise with *σ* = 0.05 are applied.

**Fig 6:**
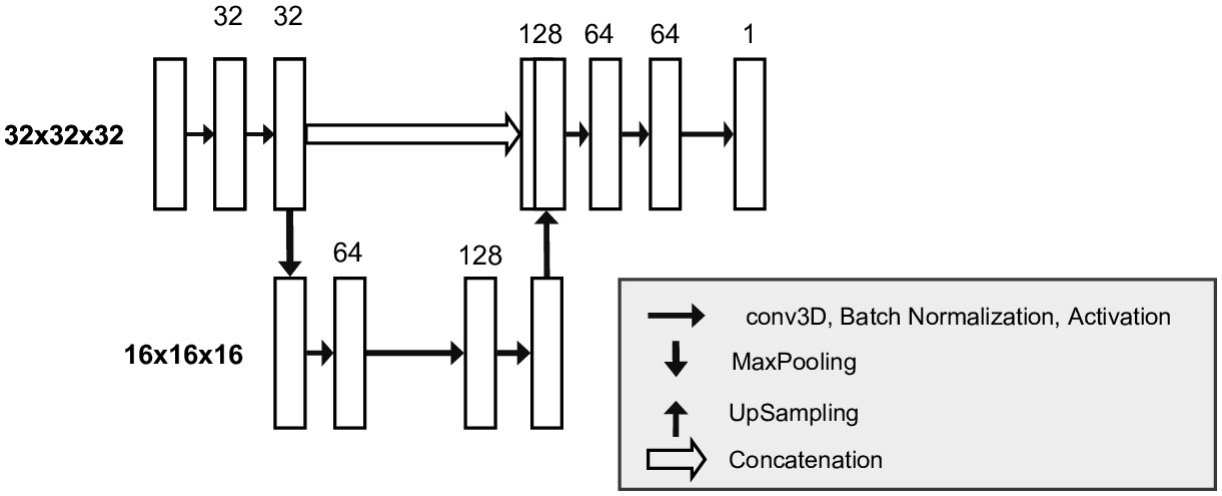
The shallow architecture of the U-Net model with a two level encoding and decoding allowing to segment 32 × 32 × 32 patches (adapted version of Fig 1 of Ronneberger et Al. [13]).

Based on this enlarged training dataset, a 3D U-Net model is trained as shown in Fig 6 within 100 epochs using an early stopping strategy [43] with patience of 30 epochs and a minimal quantity change rate of 0.0001, an Adam optimizer [44] with a learning rate of 0.0001 and a Sigmoid activation function [45]. As proposed by Fu et Al. [46], this model consists of a shallow architecture with 831,105 parameters, allowing to reduce resource costs with only low performance losses. Like this, the model consists of an encoding path and a decoding path, with only two levels each. At each of these levels, multiple three-dimensional convolutions with a kernel *k* = 3 × 3 × 3 and a stride *s* = 1 × 1 × 1 are applied, together with a batch normalization and a Rectified Linear Unit (ReLU) activation function. These steps are followed by a max pooling step on the encoding path, respectively an up sampling step on the decoder side. Due to these steps, the resolution and depth of the patches are reduced to half after the encoder and doubled after the decoder. The resulting prediction thus generated is finally reshaped according to the input. The model is trained using a binary Focal Loss optimizer [47] with *γ* = 2. This is done, because of the extreme foreground-background class imbalance within the image stacks with a background portion between 99.865% to 99.963% versus a vessel portion between 0.036% to 0.134%. Using this optimization function, more focus is put on the misclassified vessel examples during training.

**Fig 7:**
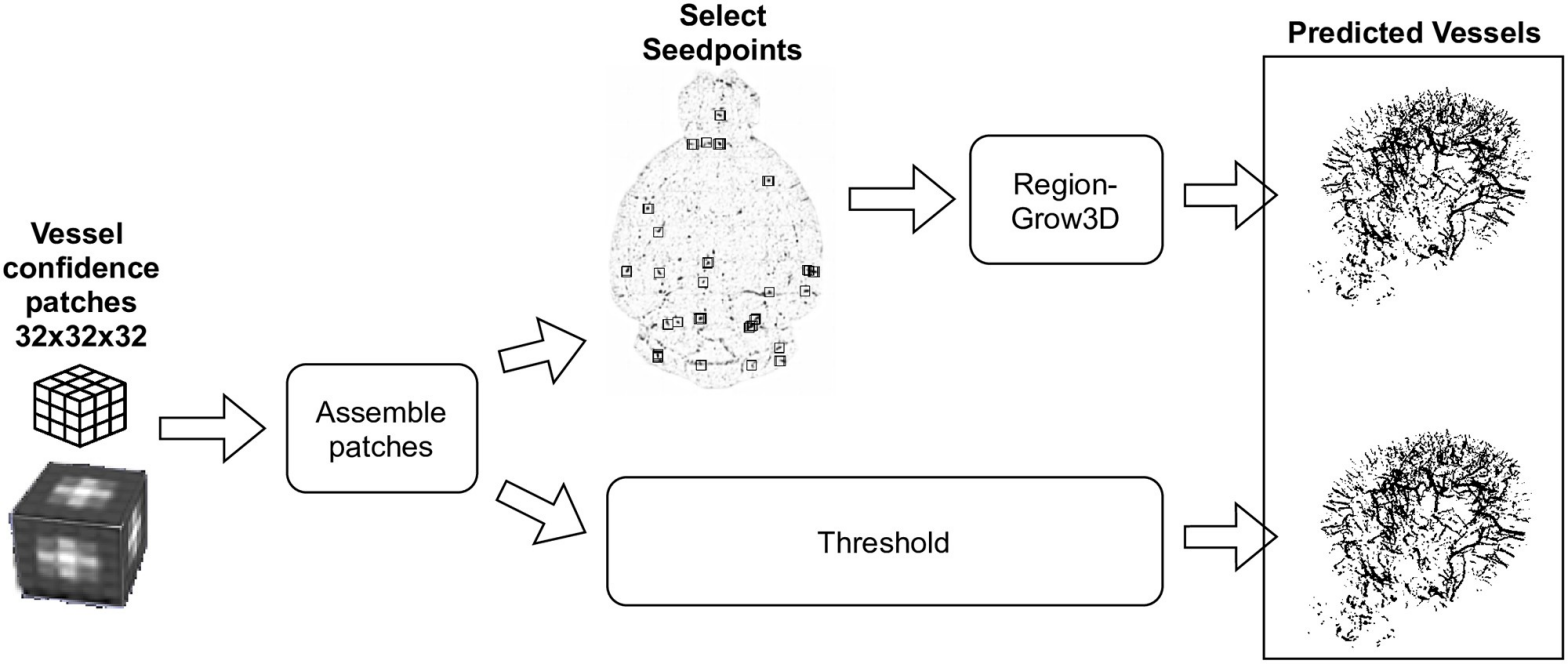
The two evaluated post-processing methodologies, namely the threshold and the region growing approaches, which are applied after the model prediction using the trained U-Net model to get the actual predicted vessels.

Due to the situation, that the model is trained using patches with a size of 32 × 32 × 32 voxels, the splits have to be reassembled after the model prediction based on the obtained patch ordering. The reassembled result consists of vessel probabilities per voxel and have to be post-processed (c.f. Fig 7). To do so, two different post-processing methodologies are evaluated. On the one hand, a simple threshold approach is used and on the other hand, a three-dimensional region growing process is used. The former one is based on the automatic selection of the most significant voxels with a threshold value *s* as seed points, which in turn are used together with a threshold value *t* for the actual growing process. The whole approach is described as pseudocode in Algorithm 1.

**Algorithm 1** Pseudo algorithm describing the proposed approach including the pre-processing steps, the actual segmentation and the post-processing.

**Require**: *σ* > 0; *t* > 0; *s* > 0; *useRegionGrowing*

 *x* ← *readImageStack*()

 *y* ← *readBrainMask*()

 *z* ← *cropImageUsingBrainMask*(*x*, *y*)

 *z* ← *applyPadding*(*z*)

 *z* ← *applyGaussianBlur*(*z*, *sigma*)

 *z* ← *normalize*(*z*)

 *patches* ← *separatePatches*(*z*)

 *model* ← *loadUnet*()

 *processed* ← *list*()

 **while** *notEmpty*(*patches*) **do**

  *patch* ← *pop*(*patches*)

  *pred* ← *fit*(*model*, *patch*)

  **if** useRegionGrowing **then**

   *seeds* ← *threshold*(*pred*, *s*)

   *pred* ← *regionGrowing*(*pred*, *seeds*, *t*)

  **else if** not useRegionGrowing **then**

   *pred* ← *threshold*(*pred*, *t*)

  **end if**

  *append*(*processed*, *pred*)

 **end while**

 *result* ← *reassamble*(*procssed*)

### Implementation and setup

The proposed methodology is implemented using Python 3.8 and primarily using the Tensorflow framework in version 2.3.0 with its Keras 2.3.0 backend. For comparison, the Frangi vesselness filter implementation of Sci-Kit image in version 0.18.3 is used. All implementation details and used datasets are open-sourced on GitHub and Zenodo [48] and freely available within the used Mozilla Public License 2. The proposed U-Net models are trained and all methods are evaluated on a Windows 10 system with an AMD Ryzen 9 3900 X 12-Core processor with 64 GB RAM and a Gigabyte GeForce RTX 2070 SUPER.

## Results

The proposed methodology is evaluated using a leave-one-out cross validation approach [49]. For this, the model is trained eight times based on a sub dataset consisting of seven image stacks from the base dataset. The remaining image is used for the evaluation of the model accuracy in comparison to the known ground truth. The training of the segmentation models takes 5259.9 seconds in average with a standard deviation of 117.13 for 100 epochs using the mentioned hardware setup.

Based on these raw results, the proposed post-processing methodologies are evaluated using different setups with *t* = [0.3, 0.45, 0.6] for the threshold approach and *s* = [0.45;0.6] respectively *t* = [0.3;0.45] for the region growing step. In addition to that, the models are compared to the results of a hessian-based Frangi vesselness filter [14]. To maintain comparability, the input files are pre-processed in the same way for both, the U-Net based evaluation as well as the vesselness filter. The only exception for this, is the last step since the patch separation is not needed for the later approach. Instead of this step, a threshold is applied, due to the situation that a binary input is required. The thresholds are manually selected for every image stack with a value *t* = [0.02, 0.03].

The raw results of the evaluation are listed in Table 3 for the wild- and in Table 4 for the knockout-type mice image stacks, together with the used threshold value for the vesselness approach. This is done in the form of confusion matrices, which compare the actual result with the ground truth (GT) according to the true-negatives (BG, BG(GT)), the true-positives (FG, FG(GT)), as well as the false-negatives (BG, FG(GT)) and the false-positives (FG, BG(GT)). These raw results are used to calculate the actual metrics for the evaluation, namely accuracy, recall, precision and the Dice coefficient (DSC) [50], which are shown in Table 5.

**Table 3:** Segmentation results in the form of a confusion matrix comparing detected background (BG) and vessel (FG) voxels for the wild-type mice from the image stacks shown in Fig 2a to 2d using the trained U-Net model with a threshold based and a region growing based post-processing, as well as the vesselness filter with individual selected threshold values in reference to the ground truth (GT).

				Stack 1 (Fig 2a)		Stack 2 (Fig 2b)		Stack 3 (Fig 2c)		Stack 4 (Fig 2d)	
Method	Pre-(*)/Post-Processing	Parameters		BG	FG	BG	FG	BG	FG	BG	FG
U-Net	Threshold	t = 0.3	BG (GT)	78.27%	21.73%	78.80%	21.20%	78.64%	21.36%	69.45%	30.55%
			FG(GT)	0.25%	99.75%	0.05%	99.95%	0.00%	100.00%	0.00%	100.00%
		t = 0.45	BG (GT)	99.93%	0.07%	99.95%	0.05%	99.98%	0.02%	99.99%	0.01%
			FG(GT)	38.99%	61.01%	23.61%	76.39%	40.68%	59.32%	39.30%	60.70%
		t = 0.6	BG (GT)	99.97%	0.03%	99.98%	0.02%	100.00%	0.00%	100.00%	0.00%
			FG(GT)	54.81%	45.19%	43.94%	56.06%	66.91%	33.09%	63.66%	36.34%
	Region Growing	s = 0.45 t = 0.3	BG (GT)	99.85%	0.15%	99.88%	0.12%	99.96%	0.04%	99.96%	0.04%
			FG(GT)	25.81%	74.19%	11.01%	88.99%	18.71%	81.29%	21.33%	78.67%
		s = 0.6 t = 0.45	BG (GT)	99.93%	0.07%	99.95%	0.05%	99.99%	0.01%	99.99%	0.01%
			FG(GT)	40.90%	59.10%	24.80%	75.20%	46.06%	53.94%	43.65%	56.35%
Vesselness	Threshold*	t = [0.02;0.03]	BG (GT)	99.91%	0.09%	99.91%	0.09%	99.98%	0.02%	99.97%	0.03%
			FG(GT)	47.09%	52.91%	30.79%	69.21%	64.28%	35.72%	59.10%	40.90%

**Table 4:** Segmentation results in the form of a confusion matrix comparing detected background (BG) and vessel (FG) voxels for the knockout-type mice from the image stacks shown in Fig 2e to 2h using the trained U-Net model with a threshold based and a region growing based post-processing, as well as the vesselness filter with individual selected threshold values in reference to the ground truth (GT).

				Stack 5 (Fig 2e)		Stack 6 (Fig 2f)		Stack 7 (Fig 2g)		Stack 8 (Fig 2h)	
Method	Pre-(*)/Post-Processing	Parameters		BG	FG	BG	FG	BG	FG	BG	FG
U-Net	Threshold	t = 0.3	BG (GT)	78.38%	21.62%	79.42%	20.58%	78.25%	21.75%	78.06%	21.94%
			FG(GT)	0.06%	99.94%	0.16%	99.84%	0.04%	99.96%	0.00%	100.00%
		t = 0.45	BG (GT)	99.93%	0.07%	99.99%	0.01%	99.98%	0.02%	99.99%	0.01%
			FG(GT)	21.70%	78.30%	55.75%	44.25%	41.98%	58.02%	40.03%	59.97%
		t = 0.6	BG (GT)	99.98%	0.02%	100.00%	0.00%	99.99%	0.01%	100.00%	0.00%
			FG(GT)	44.84%	55.16%	73.05%	26.95%	65.89%	34.11%	64.15%	35.85%
	Region Growing	s = 0.45 t = 0.3	BG (GT)	99.81%	0.19%	99.98%	0.02%	99.93%	0.07%	99.96%	0.04%
			FG(GT)	10.14%	89.86%	37.84%	62.16%	23.24%	76.76%	20.87%	79.13%
		s = 0.6 t = 0.45	BG (GT)	99.94%	0.06%	99.99%	0.01%	99.98%	0.02%	99.99%	0.01%
			FG(GT)	23.33%	76.67%	58.50%	41.50%	45.90%	54.10%	44.06%	55.94%
Vesselness	Threshold*	t = [0.02;0.03]	BG (GT)	99.95%	0.05%	99.95%	0.05%	99.97%	0.03%	99.95%	0.05%
			FG(GT)	31.70%	68.30%	55.27%	44.73%	58.61%	41.39%	49.63%	50.37%

**Table 5:** Metric based evaluation of the raw results from Tables 3 and 4 using accuracy, recall, precision and Dice score (DSC) comparing the U-Net approach with threshold and region growing (RG), as well as the vesselness filter.

Metric	Method	Pre-(*)/Post-Processing	Parameters	Stack 1 (Fig 2a)	Stack 2 (Fig 2b)	Stack 3 (Fig 2c)	Stack 4 (Fig 2d)	Stack 5 (Fig 2e)	Stack 6 (Fig 2f)	Stack 7 (Fig 2g)	Stack 8 (Fig 2h)	Average
Accuracy	U-Net	Threshold	t = 0.3	99.81%	99.86%	99.94%	99.94%	99.76%	99.96%	99.91%	99.95%	99.89%
		Threshold	t = 0.45	99.88%	99.92%	99.97%	99.97%	99.90%	99.97%	99.95%	99.97%	99.94%
		Threshold	t = 0.6	**99.89%**	**99.92%**	**99.97%**	99.97%	**99.92%**	99.97%	**99.96%**	99.97%	**99.95%**
		Region Grow	s = 0.45 t = 0.3	99.82%	99.87%	99.95%	99.95%	99.80%	99.96%	99.92%	99.96%	99.90%
		Region Grow	s = 0.6 t = 0.45	99.88%	99.92%	99.97%	**99.97%**	99.91%	**99.97%**	99.96%	**99.97%**	99.94%
	Vesselness	Threshold*	t = [0.02;0.03]	99.85%	99.87%	99.95%	99.95%	99.90%	99.93%	99.93%	99.93%	99.92%
Recall	U-Net	Threshold	t = 0.3	**74.91%**	**89.66%**	**84.36%**	**81.18%**	**90.20%**	**64.93%**	**79.06%**	**81.61%**	**80.74%**
		Threshold	t = 0.45	61.01%	76.39%	59.32%	60.70%	78.30%	44.25%	58.02%	59.97%	62.24%
		Threshold	t = 0.6	45.19%	56.06%	33.09%	36.34%	55.16%	26.95%	34.11%	35.85%	40.34%
		Region Grow	s = 0.45 t = 0.3	74.19%	88.99%	81.29%	78.67%	89.86%	62.16%	76.76%	79.13%	78.88%
		Region Grow	s = 0.6 t = 0.45	59.10%	75.20%	53.94%	56.35%	76.67%	41.50%	54.10%	55.94%	59.10%
	Vesselness	Threshold*	t = [0.02;0.03]	52.91%	69.21%	35.72%	40.90%	68.30%	44.73%	41.39%	50.37%	50.44%
Precision	U-Net	Threshold	t = 0.3	38.08%	47.61%	36.75%	40.23%	34.88%	52.47%	38.04%	39.84%	40.99%
		Threshold	t = 0.45	52.41%	65.15%	58.39%	63.03%	59.51%	74.10%	59.17%	63.60%	61.92%
		Threshold	t = 0.6	**64.27%**	**78.72%**	**74.84%**	**79.36%**	**79.25%**	**86.18%**	**75.81%**	**78.25%**	**77.09%**
		Region Grow	s = 0.45 t = 0.3	39.92%	49.58%	40.05%	43.76%	39.30%	57.85%	40.26%	43.72%	44.31%
		Region Grow	s = 0.6 t = 0.45	54.27%	67.14%	60.87%	66.85%	63.61%	78.23%	62.87%	67.11%	65.12%
	Vesselness	Threshold*	t = [0.02;0.03]	42.33%	48.31%	33.96%	34.46%	62.74%	27.24%	41.81%	25.73%	39.57%
DSC	U-Net	Threshold	t = 0.3	50.50%	62.20%	51.20%	53.80%	50.31%	58.03%	51.37%	53.54%	53.87%
		Threshold	t = 0.45	56.38%	70.32%	**58.85%**	**61.84%**	67.63%	55.41%	**58.59%**	**61.73%**	**61.34%**
		Threshold	t = 0.6	53.07%	65.49%	45.89%	49.85%	65.05%	41.06%	47.05%	49.17%	52.08%
		Region Grow	s = 0.45 t = 0.3	51.91%	63.68%	53.66%	56.24%	54.68%	**59.93%**	52.82%	56.32%	56.16%
		Region Grow	s = 0.6 t = 0.45	**56.58%**	**70.94%**	57.20%	61.15%	**69.53%**	54.23%	58.16%	61.02%	61.10%
	Vesselness	Threshold*	t = [0.02;0.03]	47.03%	56.90%	34.82%	37.40%	65.40%	33.86%	41.60%	34.07%	43.88%

Next to the segmentation results, also time measurements have been carried out for the U-Net based and the Vesselness segmentation approach, as well as for the two post-processing steps. These measurements were done for all eight image stacks and the average performance results are shown in Table 6.

**Table 6:** Average performance measurements and the associated standard deviation of the segmentation methods applied to the eight image stacks using the proposed U-Net model and the state-of-the-art Vesselness filter, as well as for the used post-processing methods. The shown values are in milliseconds.

Method	Segmentation		Post-Processing	
	U-Net & Patch (Dis-)Assembling	Vesselness	Threshold	Region Growing
Time	13149.64 ± 549.3	158716.18 ± 1174.47	117.12 ± 2.51	728.19 ± 87.15

## Discussion

The results listed in Table 5 show that the utilization of a shallow 3D U-Net model is an improvement to the state-of-the-art vesselness filters also in the case of a small reference dataset. This is illustrated by the better average values of the U-Net approach in comparison to the vesselness filter in accuracy (99.95% and 99.92%), recall (80.74% and 50.44%), precision (77.09% and 39.57%) and Dice score (61.34% and 43.8%). With this, the recall shows that about 81% of all vessels are detected successfully at a precision of 77%, using the proposed U-Net based approach. According to the individual results, the U-Net based approach is able to detect vessels more reliable for all the image stacks within the created dataset. The Dice score also shows that the used post-processing methodology has only small influences to the final result. Both, the threshold-based and the region growing based approaches show similar characteristics with only small differences in the decimal place. Additionally, Table 6 shows that the U-Net based approach is also faster compared to the vesselness filter including the required patch dis- and also reassembling, also when using the more-complicated Region Growing post-processing method.

Next to the advantages, there are of course limitations. As indicated by the related work, much higher accuracies can be reached using on the one hand bigger data sets, but on the other hand also larger segmentation models as proposed by Hilbert et Al. [51] or Tetteh et Al. [52] Since the amount of available data is highly limited for pre-clinical studies, this was not in focus of our work, resulting in comparable lower Dice scores.

## Related work

There is nearly no related work, to the best of the authors’ knowledge, that describe brain vessel segmentation methods in the context of mice *μ*MRI datasets and even less in the context of preclinical studies. Comparable publications mostly show different approaches in the context of human brain vessel segmentations (c.f. the review by Chen et al. [53]), such as Hilbert et Al. [51]. In this publication, a deep learning approach for arterial brain vessel segmentation for patients with cerebrovascular disease is presented. The authors introduce an extended three-dimensional U-Net based model architecture, called BRAVE-NET. This model architecture composes the classic U-Net architecture with two additions—namely a context aggregation and a deep supervision step. Using this methodology, the authors reach a Dice coefficient of 93.1% within the presented evaluation. Like in the presented work, the input stacks are separated into smaller patches, but with a different size of 64 × 64 × 8 voxel. The publication highly differs from the present work according to the used dataset. On the one hand, the dataset contains both ToF as well as MRI stacks with different sizes of 312 × 384 × 127 voxels and 644 × 768 × 136 voxels of human brains. On the other hand, the dataset also highly differs in the number of sample data with 264 image stacks for the BRAVE-NET approach and only eight image stacks within the present work.

Next to that, also Tetteh et Al. [52] present an approach for segmenting blood vessels within three-dimensional angiographic brain volumes using machine learning approaches. The authors present different variants of their so called DeepVesselNet architecture based on (I) a full convolutional network using four convolutions and a sigmoid classification, (II) a U-Net based architecture and (III) a V-Net based architecture. Both, the U-Net as well as the V-Net based architectures are adapted by replacing the 3D convolution layers by cross-hair filters. The proposed architectures are compared against each other, but also with two classic U-, respectively V-Net models. For the training of the models, three different datasets have been used. First of all, a synthetic dataset, that consists of 136 image stacks with 325 × 304 × 600 voxels is used. Twenty image stacks are selected from this dataset for the evaluation of the developed methodology. Next to that, a ToF, respectively a Magnetic Resonance Angiography (MRA) dataset with 40 clinical images with a size of 580 × 640 × 136 voxels is used. This dataset is split with a 50% to 50% ratio for the evaluation. Finally, a micro computed tomography angiography (*μ*CTA) dataset is used. In contrast to the first two datasets, the last one consists out of image stacks of 20 rat brains with a resolution of 256 × 256 × 256 voxels, that is extended with four additional image stacks for the evaluation. Using the presented methods, the authors are able to reach a Dice coefficient of 99.86% for the synthetic, 86.68% for the ToF and 96.27% for the *μ*CTA dataset. In contrast to the present work, the authors of the DeepVesselNet architectures again focus on a segmentation approach in the context of comparable bigger datasets without the limitations of a small reference dataset. Unlike, Hilbert et Al. [51] the presented DeepVesselNet is not only applied for human image stacks, but also for rat brains and is more comparable to the present work.

Next to the already mentioned publications, also Poon et al. [54] addressed the automatic segmentation of cerebrovasculature structures of mice and rats. Comparable to our work, the authors make use of a U-Net model to segment vessels. However, there are major differences between both methodologies. Foremost, Poon et al. are using an image dataset, that consists of 70 3D image volumes created using two-photon fluorescence microscopy. This highly differs from our image dataset with only eight 3D *μ*MRI image stacks. Next to that, the authors rely on a deeper U-Net architecture based on five levels with 16 to 256 convolution filters applied. This model architecture also only applies 2D filters and like this does not make use of three-dimensional connections in the volumetric data.

Finally, also Li et al. [55] have addressed the topic of automated segmentation of cerebrovasculature structures using deep learning methodologies. In contrast to the other works, the authors do not only use convolutional neural networks such as a U-Net, but used a combined architecture together with a Swin vision transformer [56]. The Swin vision transformer is used as feature extraction blocks within the encoder of the U-Net architecture. For the development of their approach, the authors used 282 volumes from three different datasets that were all created using Micro-Optical Sectioning Tomography (fMOST). The datasets were manually annotated using the Amira software, comparable to our procedure. Using their adapted transformer architecture, Li et al. are able to reach a Dice score around 96%—97% for the three used data sets. In conclusion, the authors state that, traditional CNN-based methods offer certain advantages in handling image details compared to their complex architecture.

To summarize the differences of the mentioned publications: Previous work has been conducted in the field of vessel segmentation. But these publications are hardly comparable to our work, because of three main reasons: (I) The authors focused on the segmentation accuracy based on huge data sets with dozens to hundreds of images, while we only use a small set of eight images. (II) The image modalities are highly different with MRI, ToF or *μ*CTA datasets compared to *μ*MRI such as in our case. Finally, (III) the authors mainly focused on the segmentation in human brains, showing greater vessel diameters and lengths then for mice. For this reason, the results shown in our publication can only hardly be compared to those of the other authors, except of the general methodology of using a U-Net like model architecture. However, the model architecture also shows big differences in detail. Because of the relatively limited dataset, we have opted for a shallow U-Net model design, which consists of just 831,105 trainable parameters. In comparison to other architectures, this number of parameters is significantly lower and for this avoids overfitting and improves generalization as discussed by Brigato and Iocchi [57]. For instance, the classic U-Net architecture with four layers has approximately 6 million parameters [51]. The BRAVE-NET architecture has around 10 million parameters [51], the DeepVesselNet-UNet [52] has approximately 4 million parameters, the DeepVesselNet-VNet has about 17 million parameters [52], and the VNet architecture has roughly 23 million parameters [52]. Finally, The Swin transformer is available in three versions with 29 million, 50 million and 88 million parameters [56]. Li et al. [55] do not state, which model is used in their combined U-Net architecture.

## Conclusion

Many (pre-)clinical computer vision domains show a lack in training data. In the present work, the utilization of a machine learning based approach for the segmentation of brain vessels in mice *μ*MRI scans using a small reference dataset is successfully shown and addresses the first research question *RQ*_1_: *Are deep learning architectures applicable for the segmentation of mice brain vessels using a small μMRI reference dataset?*. The influences of post-processing methods like thresholding or region-growing showed only small influences in the final result, which addresses *RQ*_2_: *Which influence do different post-processing methods have?*. It is shown that the presented methodology is able to outperform classic state-of-the-art methodologies in the form of vesselness filters, resulting in improved vessel detection rates represented by a Dice coefficient of 61.34%, without the need for neither an additional user input nor many training datasets. Based on this results, also the final research question *RQ*_3_: *How does a deep learning based methodology perform compared to classic approaches such as vesselness filters?* is successfully addressed within this work. In summary, a major strength of the presented approach is that it succeeds even for a small ensemble of datasets—and hence represents a practical, fast and open-source solution [48] to many preclinical research projects that are usually limited in the number of mice or rats. If additional image stacks are acquired during preclinical studies, the re-training of the created models allows to further improve the results and like this to get a first pre-segmentation that can be improved step by step.

## Supporting information

S1 Appendix (PDF)
