# Supplementary material for: U-Net based vessel segmentation for murine brains with small micro-magnetic resonance imaging reference datasets
Source: PLoS One. 2023 Oct 12;18(10):e0291946. doi: 10.1371/journal.pone.0291946 (PMC10569551; doi:10.1371/journal.pone.0291946)
Supplement: S1 Appendix — (PDF) [file pone.0291946.s001.pdf]

Appendix

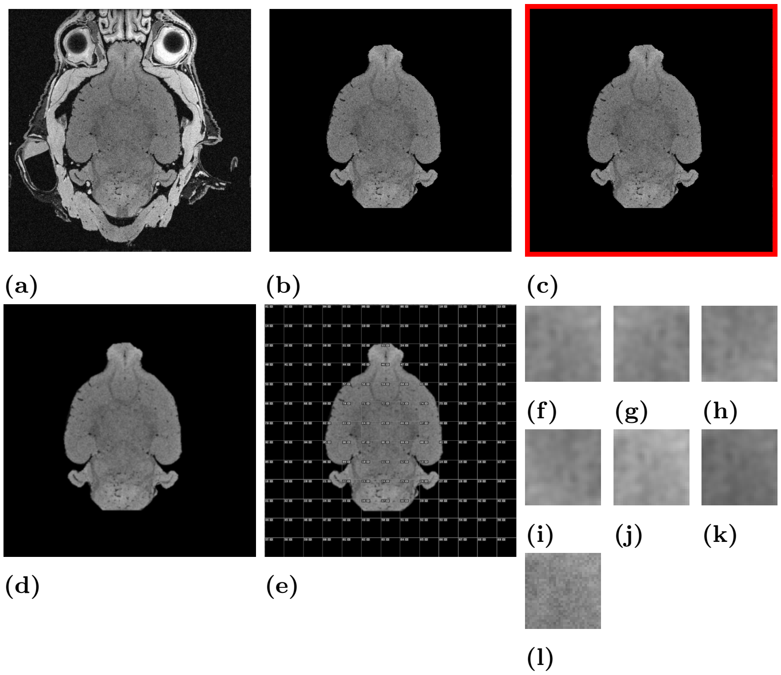

Fig 8. This figure shows the individual results after every step of the pre-processing pipeline. It is based on one 400×400 pixels sample layer (a) of the image stack from Figure 1 with (b) showing the cropped brain based on the given brain mask, (c) the padded version with a size of 416×416 pixels (red color is only used for visualizing the additional area, which is actually black), (d) the blurred version using a Gaussian blur with $\sigma = 0.5$ (refer to zoomed-in digital version to see differences) and (e) the marked 169 patches with a size of 32×32 pixels each. Figure (f) shows samplewise one layer of a patch that is augmented using randomly applied methods such as (g) horizontal flip, (h) vertical flip, (i) diagonal flip, (j) brightening up, (k) darkening and (l) applying a noise.
